# IMCI and ETAT integration at a primary healthcare facility in Malawi: a human factors approach

**DOI:** 10.1186/s12913-018-3803-5

**Published:** 2018-12-29

**Authors:** Sarah Kathryn Robertson, Kristina Manson, Evridiki Fioratou

**Affiliations:** 1 Medical School, University of Dundee, Dundee, Scotland; 2 East Lancashire Hospitals Trust, Lancashire, England

**Keywords:** IMCI, ETAT, Human factors, International health

## Abstract

**Background:**

Integrated Management of Childhood Illness (IMCI) and Emergency Triage, Assessment and Treatment (ETAT) are guidelines developed by the World Health Organization to reach targets for reducing under-5 mortality. They were set out in the Millennium Development Goals. Each guideline was established separately so the purpose of this study was to understand how these systems have been integrated in a primary care setting and identify barriers and facilitators to this integration using a systems approach.

**Method:**

Interviews were carried out with members of staff of different levels within a primary healthcare clinic in Malawi. Along with observations from the clinic this provided a well-rounded view of the running of the clinic. This data was then analysed using the SEIPS 2.0 work systems framework. The work system elements specified in this model were used to identify and categorise themes that influenced the clinic’s efficiency.

**Results:**

A process map of the flow of patients through the clinic was created, showing the tasks undertaken and the interactions between staff and patients. In their interviews, staff identified several organisational elements that served as barriers to the implementation of care. They included workload, available resources, ineffective time management, delegation of roles and adaptation of care. In terms of the external environment there was a lack of clarity over the two sets of guidelines and how they were to be integrated which was a key barrier to the process. Under the heading of tools and technology a lack of guideline copies was identified as a barrier. However, the health passport system and other forms of recording were highlighted as being important facilitators. Other issues highlighted were the lack of transport provided, challenges regarding teamwork and attitudes of members of staff, patient factors such as their beliefs and regard for the care and education provided by the clinic.

**Conclusions:**

This study provides the first information on the challenges and issues involved in combining IMCI and ETAT and identified a number of barriers. These barriers included a lack of resources, staff training and heavy workload. This provided areas to work on in order to improve implementation.

## Background

The Millennium Development Goals (MDGs) were developed in 2000 and adopted by 189 heads of state worldwide with the intention of tackling poverty, hunger, illness, gender inequality and lack of access to education and clean water [1]. The intended deadline for these goals was 2015. Although the under-5 mortality rate was more than halved between 1990 and 2015, reduced from 91 to 43 per 1000 live births the target of a two thirds reduction was not met on a global scale. Malawi as an individual country did, however, meet the target of two thirds reduction set in the MDGs [2]. Approximately 70% of childhood deaths globally are caused by one or more of malaria, measles, malnutrition, diarrhea and acute respiratory infections [3]. The overlapping diseases cause complications for health care workers in terms of both diagnosis and treatment of disease. Coming to a single diagnosis is difficult, often impossible, especially when there are limited resources available for investigations.

To meet the goals outlined in the MDGs and reduce deaths due to preventable disease in children, the WHO and UNICEF developed the Integrated Management of Childhood Illness (IMCI) system. This is aimed at qualified doctors, nurses or health care workers and provides a framework for deciding treatment routes for children who present to the clinic. It focuses on the most common causes of preventable infant death. The strategy was first implemented in 1995 whereby a more holistic approach was adopted to under-5 s care [4]. As more than one of the common preventable diseases are often found in one patient it aims to bypass diagnosis of individual diseases and instead classify the patient depending on how urgently they require treatment. To identify the common preventable diseases all patients should be checked for the four main symptoms: cough or difficult breathing; diarrhea; fever; and ear problems. This, along with assessing the child’s nutritional and immunization status, allows the illness to be classified and then treated systematically [3]. Other important aspects to IMCI care are health education for the guardian and giving follow up care. IMCI was designed for use in countries with an infant mortality rate of 40 per 1000 live births or greater [4, 5].

Malawi is one of more than 100 countries where IMCI is central to child health efforts. The healthcare system in Malawi benefits from a network of clinics provided by multiple sources. These include the Malawian government (providing around 28% of funding), private companies (16% of funding) and charitable organisations (56% of funding), such as the Christian Health Association of Malawi (CHAM) [6, 7]. From 2006, 44 of 46 Sub-Saharan African countries have initiated implementation of IMCI and in Malawi the current coverage is over 50% of the countries’ districts [8]. Studies based in Tanzania and Bangladesh have shown that the use of IMCI guidelines improved the quality of care provided without a significant increase in cost [9]. Progress is steady in Malawi, the 2015 under-five mortality rate was recorded as 61 deaths per 1000 live births, declining from 242 in 1990 [10]. Though this is impressive progress, it is not low enough to meet the new target in the U.N. Secretary General’s Global Strategy for Women’s, Children’s and Adolescent’s Health (2016–2030). This aims to have all countries reduce their under-five mortality to 25 or less per 1000 live births by 2030 [11]. There has been plenty of research into how IMCI has contributed to the steps taken towards meeting the Millenium Development Goals [12–14]. Due to a high disease burden and critical human resource shortage, Malawi still faces challenges to sufficient IMCI implementation. In order to attempt to offset the human resource shortage a new cadre of health care worker has been created by the Malawian Ministry of Health. These are the health surveillance assistants (HSAs). This group now comprises 30% of the countries health workforce [15]. They are community health workers, with a minimum training requirement of 8 weeks from the Ministry of Health [16]. Frustration at this lack of training and their increasing responsibilities and lack of supervision is widely reported amongst HSAs [15–17].

Developed in Malawi, “Emergency Triage, Assessment and Treatment” (ETAT) was a programme created by the WHO to complement IMCI. Published in 2005 [3], the package is designed to implement triage and management of commonly occurring emergencies. In the primary care setting, ETAT is broken down to adapt to the non-clinical health worker level, i.e. HSAs, specifying that only the emergency triage (ET) component applies here. [18] It is intended that anyone in the healthcare environment, right down to the janitors, should be able to identify the children most in need of treatment and know where to send them.

Although the IMCI and ETAT programmes are intended to be used collaboratively [19, 20] there is little knowledge of how the two packages interact or guidance on how they are expected to be used together. Each guideline was established separately so the purpose of this study was to understand how these systems have been integrated in a primary care setting and identify barriers and facilitators to this integration using a systems approach.

## Methods

This cross-sectional study intends to analyse the integration of these two programmes by focusing on a primary healthcare clinic in Malawi and using a human factors approach. Adopted from the engineering profession, this approach examines the systems, processes and conditions of an environment with the intention of minimizing the room for human error [21, 22]. By scrutinizing the circumstances that have led to previous mistakes future errors can hopefully be prevented. Areas for improvement may be found by simplifying or standardizing procedures, changing the way devices are used, improving communication between teams or building in opportunities to identify and recover from mistakes [22]. The specific framework used was the SEIPS 2.0 model (Systems Engineering Initiative for Patient Safety). This advocates that the appropriate solution to problems in the healthcare system can only be found if the entire system is considered [23]. It offers a structure to identify the influencing factors in the system, which can be used with regard to the primary healthcare clinic to analyse how well the IMCI and ETAT systems have been adopted and can work together. The use of a cross-sectional study gives a clear picture of this clinic at a particular moment in time. This information about how this clinic is functioning while trying to implement both systems will give an idea of how well the systems work together. Hopefully it will also provide data for further hypotheses about which barriers could be altered in order to improve this functioning and further decrease the under-5 mortality rate at this clinic and others of its kind.

### Context

This research was conducted at a primary health care facility located outside Blantyre, Malawi. Blantyre is the second most populous city of Malawi and is the financial capital [24]. It is an urban area serviced by one public hospital, 6 private hospitals and one specialist orthopaedic hospital run by a non-governmental organization (NGO) [6]. The centre at the focus of this study is one of 6 centres run cooperatively by the local council and District Health Office (DHO). It had a catchment area of 158,000 people, 17% of which were under-5. It was deemed appropriate to fulfil the exploratory nature of this study, due to the limited knowledge concerning previous evaluations of IMCI in that area.

### Participants

To be eligible for the study participants had to be staff members dealing with assessment of under-fives. These included Medical Assistants (MAs), Student Nurses (SNs) and Health Surveillance Assistants (HSAs). Selecting staff from various cadres allowed a broader insight to the perceptions of child assessment in the clinic. The staff members suitable were suggested by the contact at the clinic prior to the interviewer arriving at the clinic. A key selection prerequisite was the English language proficiency of staff members. This was unavoidable due to the interviews and analysis being carried out in English by English speakers. Eleven interviews were completed, five with MAs, three with SNs and three with HSAs. This selection covered the range of cadres working at the clinic as there were no doctors available to treat patients there.

### Procedure

Semi-structured interviews were conducted to elicit in-depth accounts of the IMCI process as experienced by staff. This format was selected to ensure that there was enough structure to the interviews to allow them to be comparable but also allow enough flexibility to incorporate different staff levels without generating bias. Open questions were used to allow in-depth insight into the perspectives of participants. Interviews were recorded on a digital recorder and lasted between 25 and 49 min. Interviews were transcribed verbatim by the researcher. Thematic analysis [25] was used to explore the interview data. An inductive approach was applied using the SEIPS 2.0 Human Factors framework [26]. This framework was chosen in order to highlight the system design in place and also how this design influences other connected processes within the clinic. [27]The intention was to show how the two procedures together were influencing the running of the clinic. This data, along with informal observations from the clinic, was used to construct a process map, detailing the flow of patients through the clinic and when and why they interact with different members of staff. The informal observations were carried out by the researcher within the clinic. In addition to conducting interviews the researcher sat in on clinics to see for themselves how the guidelines were implemented.

Prior to commencing the study, ethical approval was given from the University Research Ethics Committee in Dundee and from the Blantyre District Health Office.

## Results

### Process mapping

**Fig. 1:**
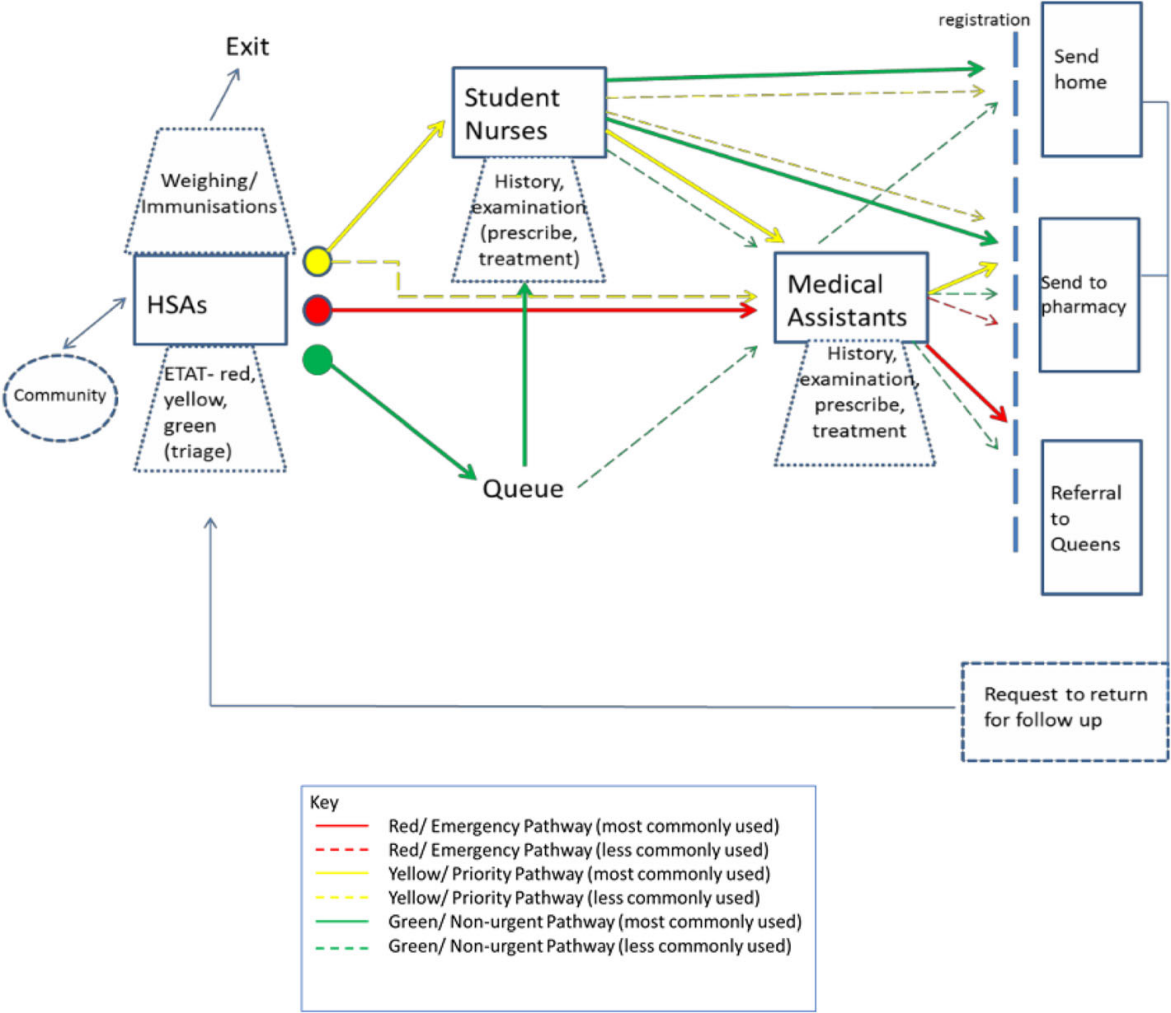
Process Map of the Primary Healthcare Clinic in Malawi

Through interviews and informal observations around the facility a process map was developed. It shows the flow of patients through the clinic and highlights the roles of different staff members. It gives some understanding of the work system by contextualizing the staff present at the clinic and the roles they undertake. The process map (Fig. 1) depicts the different levels of staff members in solid rectangular boxes. Their roles are then outlined in the dotted rhombus box. The coloured lines represent the triaging as outlined in the ETAT scheme. The solid lines refer to the most common route and the dotted lines show other options that were utilized less often. This is specific to the clinic where the interviews took place. On admission to the clinic patients were greeted by HSAs who assess them for emergency signs (red pathway), priority signs (yellow pathway), or if these are not present they are classified as non-urgent (green pathway) [28]. This classification is the basis of the ETAT system, and it was intended that anyone should be able to carry this out even if they had no qualification to treat patients. Their other roles in the clinic included weighing and immunisations, which was done for both sick and well children. They were also responsible for health education within the community. These jobs are important roles in the IMCI guidelines and are to be carried out by healthcare professionals.

Student nurses saw patients who were classified as a priority first while other, non-urgent patients, waited in a queue. The student nurses took more in-depth histories from the patients and their guardians and were able to administer some treatments. These patients could then either be discharged or passed on to the MAs. Student nurses also took part in immunisation and weighing processes and offered health education to the guardians of the patients they saw.

Patients who had emergency signs were taken immediately taken for assessment by MAs. This was a very brief assessment that paid attention to danger signs. Their outcome was decided and any necessary treatment was administered before either transferal to the nearest hospital, or discharge to the pharmacy or home with a possible request for follow up care, at which point the patient was returned to the beginning of the system.

### SEIPS 2.0 work system elements impact on the clinic

As summarised in Table 1, the SEIPS 2.0 model was used to explore the interview data, revealing subthemes of the interview content. These were then categorised as either barriers and facilitators to efficiency of the clinic.

**Table 1:** Work system elements that impact on the Clinic as reported by staff

SEIPS 2.0 Work system elements	Subthemes from the Interviews	
	Barriers	Facilitators
1. Organisation	Workload Availability of resources Time management Work schedules Adaptations to care	Training Prioritisation
2. External environment	Transportation Governmental factors	Education Implementation of programmes
3. Internal environment	Cleanliness Space in facility	
4. Tools and technology	Guideline tool availability Usability of job aids	Importance of health passport Centralised recording
5. Person	Teamwork Staff personal motivations Self/colleague confidence Pre-existing patient factors Care dependent patient factors	Teamwork Staff personal motivations

### Organisational barriers/facilitators

#### Workload

Over half of the interviews identified the workload as a major organisational barrier to effective child assessment. All five of the MAs interviewed were included in this group. Shortage of staff and overcrowding were noted as contributors to reducing the efficiency of care. There was awareness that the workload itself should not be regarded as the main barrier, but rather the facilities and staff available to deal with the number of patients was lacking.

*“Maybe the challenges, but maybe the overcrowding, yeah maybe we fail to manage all the clients at the one time. There are a lot of them.”(SN)*

*“… the right way to put it is shortage of staff…. But then if we have good numbers we can have managed them appropriately.” (MA)*

#### Availability of resources

This was described by nearly all participants as a key problem in the healthcare centre. Resources include medication, equipment and vaccinations. Another key area of concern was the ability to test and therefore diagnose malaria. With only one microscope, reliance was on malaria rapid tests which were in short supply. Lack of resources were a key influence on the referral process, as many people were sent away in search of medications from other facilities.

*“It’s not good when someone comes here and they think they have found everything they will be healed because they are going to receive drugs’, and they must go back to their home without drugs.” (HSA)*

*“because of lack of some of the resources it has become a problem. That’s why we refer people…all that we need are resources.” (SN)*

*“And with the changes of shortage of MRDTs, as you can see with must rely on a single microscope of which cannot microscope for all the children we have seen today.”(MA)*

#### Time management

Managing time was an issue identified by over half the interviewees. Newcomers to the clinic, the student nurses, highlighted a problem with using time effectively. Time was essentially the biggest factor delaying the child assessment.

*“I have this long queue outside there, people are screaming outside there time, time, time. I’m supposed to be seeing them at the same time I’m supposed to be seeing the child.”(MA)*

As an IMCI facilitator the concept of prioritisation was explored, with many participants speaking positively about the clinic’s ability to prioritise the cases.

*“I think the most important thing is already there. Because the prioritising the groups is very important, I appreciate that we have that system in place.”(SN)*

#### Training

Six staff members said they had undergone IMCI training. This group included all the MAs interviewed. The only other interviewed staff member who had undergone training was an HSA. Only three staff members explicitly said they had undergone ETAT training, one MA and two HSAs. One additional MA interviewee expressed a desire to become formally familiarised with ETAT.

*“At least I need official, initial training for ETAT just like my fellow clinicians underwent. So I want myself to be also trained at that.” (MA)*

Everyone reported the success of the ETAT programme currently in place, however need for training was identified by five participants. One HSA clarified that training was given to only those that were selected.

*“You are chosen to go to the training…So there are about 50 HSAs, the list comes and these 10 names they go.” (HSA)*

There was a consensus about the requirement for more training amongst staff. This was required to even out the training between different staff cadres and to ensure universal training for necessary skills.

#### Work schedules

Problems with the work schedules were identified by three of the MA participants. Concerns revolved around task sharing and other staffs’ commitment and capabilities. One MA insinuated staff members’ non-adherence to their duties could result in children not being managed effectively.

*“Because if you have seen, for example you have seen a child, a sick child who needs maybe nutritional assessment that means you didn’t get it from the ones who do it there… if they are not willing to do it that means the child is not getting the proper treatment because some other thing is missing that should be done by your colleagues there.” (MA)*

On the other hand, the benefits of having various levels of staff member adding to quality of care was also discussed.

*“Yeah it’s the best way because the HSAs helped to reduce the overload of the work.” (SN)*

#### Adaptations to care

Four out of five MAs interviewed mentioned ways they had to adapt the care provided in the clinic to combat the problems faced.

*“But for one that we see that they don’t need maybe to do all these processes, we just, we just rush and then do our work, but eh most of the times we do use this.” (MA)*

Forgetfulness on behalf of staff members led to not always checking the immunisation status of children and due to the lack of resources, the preferred treatment was often not the one actually prescribed.

*“But if we don’t have resources like malaria rapid tests, we do change our.. we maybe just prescribe an antibiotic, and we do tell them to go for the test.” (MA)*

### External environment barriers/facilitators

#### Implementation of programmes in the clinic

An awareness of IMCI guidelines was found in all five of the MAs and only one HSA. Of those that reported an awareness of the guidelines a single MA interviewee displayed a sound understanding of the concept of IMCI, while others demonstrated a failure to adopt the intrinsic “integrated” nature of the guidelines.

*“These are guidelines where by, mmm a sick child is interviewed and assessed wholly, maybe in short that is what I can say. It’s like…. when we say integrated. We use all the angles of a sick child, in a package.” (MA)*

All participants were aware of the key prioritising concept of ETAT. Participants could explain the roles of the important components of the programme to hasten the triaging and treatment process for sick children. The ETAT procedure mainly concerned the HSAs and their interactions with patients prior to taking the child to higher level members of staff. Based on the descriptions of the roles of different staff members, the program only encompasses pre-MA assessment.

*We have got three ruler we have got to assess the child, the first ruler is the red one… that one is emergency, that one will not stay for very long time, we just take them there to consultation room…. The second ruler here is yellow, that one is priority, which means they are sick, they are very sick, not an emergency, maybe suffering from malaria, maybe a temperature not below 38 degrees. Yeah that one is priority. So we take that one straight to the front to see the doctor. The third one is the green ruler. They are sick but not very sick. They can stay on the line and squeeze, before they see the doctor.”(HSA)*

#### Evolving guidelines

The data raised queries on the implementation of programmes in the clinic. Only one participant appeared to have perspective of the evolving process. Some participants displayed a misinterpretation of the interaction between the two programmes, with one believing that ETAT was replacing IMCI completely rather than function simultaneously and another thinking it wasn’t relevant to them at all.

*“ IMCI… had started and it was flowing but now there’s another programme that has crept into the system, and that is ETAT now. ETAT has some things that concern IMCI, but only some time, ETAT… systematically is killing the IMCI programme.”(MA)*

*“They say that this one, health centres in urban areas, but as of this time they are not included in IMCI. This time they are just looking at rural areas.” (HSA)*

#### Transportation

Transportation was noted as a problem by five participants. In particular, poor ambulance services were found to greatly inhibit the referral process. In addition to patient transfers two HSAs identified transport as an issue for their work in the community.

*“Because we advise these patients to board local transport because we can’t rely on these ambulance to take patients to Queens.” (MA)*

*“outreach clinics…that’s the problem we have we don’t have the transport there, because we do immunisations, we need to take everything to go there. So it’s difficult to carry it.”(HSA)*

#### Education

Patient Education drives were identified across all cadres as an important factor in under-five care. HSAs noted the importance of community mobilisation through education.

*“We explain to them the goodness of going to the hospital. They understand, and they come….I think mobilisation is needed to tell them the goodness of the, the goodness of coming to the hospital early.” (HSA)*

An SN was able to give an example of the kind of information they routinely imparted on their patients and guardians.

*“If the child looks healthy, everything is ok, we encourage the mother to continue, but maybe if the child is having a history of malaria, we see in the health passport malaria, malaria, malaria….we sit down with colleagues and asking the mother what she think about the history of malaria. Maybe there might be the problems they are having at home maybe they aren’t having mosquito nets, then you go and ask, one mosquito net for that place. Giving like health education to like cut the bushes off round the house” (SN)*

#### Governmental factors

Concerns about the role of the Government, including their adoption of the ETAT programme, were identified in half of the participants. Calling upon the government for provision of materials and staff was mentioned by four participants.

*“Because currently its being taken by the Malawi Liverpool Wellcome Trust. And it’s supposed to be passed on to the government. I don’t know if that programme will continue (laughs) sometimes they usually stop when they are handed back to the government.”(MA)*

“Also we have to, it’s like cry for the government, they need to make sure the health centres is having vaccines something like that. And nutrition…”(SN)

### Internal environment barriers/facilitators

#### Space available in facility

Three participants identified space in the facility as an important factor in managing.

under-five patients. The issue of space was of particular interest when discussing health education.

*“Health education can be improved in such a way if we can have so many rooms to see patients.”(MA)*

#### Cleanliness

Two interviewees discussed concerns with the cleanliness of the environment, claiming that the level of hygiene in the facility was not conducive to optimising children’s care. They thought the cleanliness made the clinic less welcoming.

*“When I talk about the environment, its cold, it’s not very smart. Like sanitation, sanitation is cold.” (SN)*

### Tools and technological barriers/facilitators

#### Guideline tool availability

All the MAs participating identified that copies of IMCI and ETAT guidelines were lacking, impairing their ability to carry out assessments. MAs trained in IMCI were relying on memory to manage children. All staff members identified ETAT phones as being a key tool in the programme. These phones were used, for example, to communication with other health care facilities or ambulances to facilitate referrals. However, one HSA identified that these ETAT tools were not always available in the clinic.

*“Of course we are supposed to have some job aids. We are supposed to have something to check whether, I mean to check like a flow, step by step. Unfortunately we don’t have these on our desks, because most of them are worn out.”(MA)*

*“First of all we are many staff doing ETAT but problem you can say phone shortage, but staff we are ok.”(HSA)*

#### Usability of job aids

Nearly all the MAs mentioned accessibility of job aids (tools to inform management) as a limiting component to child assessment. Posters were described as a good way to make tools accessible. The usability of job aids was highlighted by two interviewees, indicating there were problems with taking responsibility for guidelines. Durability was mentioned as a problem, as was disorganization of tools. In the SN population, two participants indicated they don’t need a guide to support their practice. Conversely one out of the three student nurses disagreed with colleagues and advocated for the use of guideline tools.

*“Yeah I think we do not have enough tools. We do not have those job aids we have been talking about. Because most of them they are no longer there, most of them have got lost… At least if maybe they were improved in terms of laminating, maybe then we can keep and the can stay long.”(MA)*

*“ Yes it would help because sometimes we can remember the things without the cards, we need to have a guide to direct us, and then we can do it.” (SN)*

#### Importance of health passport

Health passports are paper booklets given to people in Malawi. The patients keep these in their own care. They contain a record of immunisations, medical conditions and medical care the patient has received [29]. The health passport was regarded as a vital tool by four participants, supporting diagnosis and prescribing as well as recognition of need for follow-up. Conversely two participants identified limitations to health passports; patients may lose them or they fall apart due to poor durability.

*” Each and every child is given a book...Apart from that in the back of that passport there’s immunisations dates...Even the weight what weigh are they supposed to be at this time.” (HSA)*

*“But the challenges, when a guardian comes in with that child we often find that that page with the immunisations it has been removed… so we take the history from the mother.”(MA)*

#### Centralised recording

Another essential tool identified was methods of centralised recording. Two HSAs identified the immunisation register as a vital means of recording. One MA explained the methods used previously to record referrals, which documented children requiring treatment at the district hospital. Nowadays the referral documentation process was non-existent.

*“When the books are lost the mothers they come again with a new book . so it’s difficult to recognise that this child has received such, such, such vaccine. So when you go back to the register you find that its BCG … yah it’s what he has received”(HSA)*

*“We used to have the form that we fill, and then we send the mum with it to (hospital) and they give us feedback…..that thinking failed because of congestion.”(MA)*

### Person oriented barriers/facilitators

#### Teamwork

Teamwork was identified as very important by all participants. The negative observations talked about coordination, communication and staff relations. Five participants from all cadres specified this as being an issue between staff of different levels. There was evidence to support the poor staff relations between team members of different levels as well as suggestions that team work was also an issue between staff of equal levels. These problems between staff members of the same cadre were specifically mentioned by MAs whereas the problems between different cadres were referenced by members of all the cadres.

*“We are supposed to work together as one team but normally, you know where, people work with different levels, there’s always problems, because we are underrated sometimes...We work together but not as one team”(HSA)*

*“One day we will be nurses, one day we may come to work at this place, which means that if that communication is poor at the beginning, I don’t expect it to be good when we come and work at this place.”(SN)*

*“….sometimes maybe because they see we are on the same level so we find it hard to listen to one another”(MA)*

Positive observations about teamwork were described by six interviewees, two of each cadre. An SN explained that coordination was taking place to enhance learning. An MA described the need for continual work to improve the coordination efforts that were already beginning to get underway. Achieving this could be done through staff continuity. There was appreciation in the clinic from some of the interviewees concerning task sharing between the roles. Despite the concerns stated previously regarding teamwork, three participants indicated sharing jobs between staff levels worked well.

*“those clinicians, medical assistants, they are very helping us and we are learning a lot from that. We a learning about the conditions the patients are having.”(SN)*

*“Yeah it’s good for the patient, because the patients can be seen with the HSAs who has less knowledge for the child, and when it comes to, I can miss something, I’m a human being as well of which a nurse can come in. It’s good.” (MA)*

#### Personal motivations

Nearly all the participants identified personal motivations that played a role in the work system. The positive features identified by eight interviewees were commitment, care towards their patients, sense of responsibility, job satisfaction, enthusiasm and being pro-active. Staff showed empathy and there was a sense of commitment to care, seen in participants who showed enthusiasm and pride in their work.

*“Team work should be there, but also all the staff, all our colleagues to have a feeling that we should help that sick child… Rather than just working because I got this job and you don’t have the, the the …Motivation to work and to do that.”(MA)*

*“I enjoy it because I don’t want to see somebody come to this hospital and die, while on the, while he or she is on the line for the doctor…And I enjoy when I see those child coming and immunised because we prevent more diseases there.”(HSA)*

The negative features described by staff consisted mainly of the opposite features to those identified as positive observations; lack of commitment to care, no sense of responsibility, lack of motivation and poor attitude. Overall the MAs and SNs were more critical. Remarks showed an impression amongst some of the participants that there was a negative attitude, and lack of commitment in some of the other members of staff.

*“We just lack the whole of it, maybe the only thing I can say we are lacking maybe, I can say that is the passion. Passion to see the children.”(MA)*

*“Yeah, there are times, people like to do their own business while they are at the hospital, instead of helping the patients they think of doing something else, maybe just sitting down.”(SN)*

#### Self/colleague confidence

Two of the participants regarded having previous clinical experiences as a foundation for their own self-confidence. Four participants, including all the participating SNs, referred to a lack of confidence in colleagues, they were unsure of the capabilities of other staff members. An SN explained that HSAs approached tasks differently, in a way which caused concern.

*“The more you meet the sick children the more you have experience because you are seeking them frequently.”(MA)*

*“there’s things we are doing but just that the HSAs , they like taking short cuts, we need to follow steps if we are doing something but they usually skip steps”(SN)*

#### Pre-existing patient factors

Pre-existing patient factors were identified as major influences on children’s management. Five interviewees expressed that a lack of understanding on the behalf of guardians’, as well as their beliefs, priorities and sense of autonomy/freedom could be barriers to health. Parents priorities were shown to influence the chance of children returning for follow up.

*“First of all maybe other woman they are refusing for me to assess the baby...Maybe the culture they have they don’t want the baby to be touched by the health workers.”(SN)*

*“But there is some other mothers who just ignore coming to this health centre for the children to have immunisations. That’s the problem we are having.”(SN)*

#### Care-dependent patient factors

Five members of staff highlighted care-dependent patient factors as an influence on management of children. Education was regarded by two MAs as key to future care. Staff members explained how patients react in the event that medications are not available. Three staff members recount the negative consequences.

*“Most people don’t have money to buy drugs, when they find here there’s no drugs, they told them they have to go to the pharmacy, so it’s difficult. So some of them, they just go back home and stay, without any education. Which is not good.” (HSA)*

*“Its either this person has to be advised to go and buy. So the person complains, and say if I had money would I even have bothered coming here? Yeah we refer them to (hospital) that say they are too tired, it’s too far. But sometimes they go.”(MA)*

## Discussion

The process mapping carried out within the clinic made the roles of staff more clear. With this information we can now examine how the process displayed here fits the intended process for each of the programmes IMCI and ETAT. Where the actual process does not fit the intended one laid out by each of these schemes possible reasons can be highlighted by the barriers and facilitators exposed using the SEIPS 2.0 framework.

Use of the IMCI system is demonstrated before patients even present to the clinic through the work of both HSAs and SNs in the community setting. The administration of vaccines and weighing showed a clear effort to prevent some of the commonest causes of infant mortality as outlined in the IMCI guidelines. If the children do present to the clinic, the vaccination and nutritional history found in their health passport can be a useful tool for triaging the patients. The health passports were identified as a useful tool but were not always used to their best advantage, commonly lost or damaged by the patients and their guardians. The immunisation register was therefore essential as a back-up tool to identify immunisations the patient had received. For example, if there is a history of a vaccine provided for certain diseases, the likelihood of this being the cause of their disease is drastically lowered. Also, previous records of weight can highlight any significant weight loss on presentation that would be a cause for concern and may indicate the severity of disease. As a result of time management issues and organizational factors, this step seems to be commonly missed in the assessment of patients. The HSAs are responsible for triaging patients who arrive at the clinic, so this is clearest area where use of the ETAT system was seen. They used the colour coding system to prioritise which patients should be seen first and by whom. This appeared to work efficiently in the case of those classified as a red, or emergency situation. They were immediately taken to see an MA. However, the route through the clinic was less clear for those with other colour classifications as there were multiple route options available. The chosen route for these patients was therefore more down to the judgment of members of staff than any specific system. This may have been due to problems within the system itself, or because of the shortage of tools available as a reminder of the system’s guidelines.

The HSAs did not display use of the treatment portion of the ETAT scheme, rendering it ET in practice at this level. This may be due to the level of training, or lack thereof that they have undertaken. The ‘emergency triaging’ aspect is also seen to a degree in the work of the SNs, to decide whether the patients require being seen by an MA also. They do perform some treatments although it is not clear whether these included those described in the ETAT guidelines, and these were often supervised by an MA anyway. This was another area where the judgement of the individual seemed to play more of a role than the deployment of any decision-making system. The role of SNs within the clinic had not been clearly defined and therefore their responsibilities and capabilities were less clear. This may have been an organizational problem at the clinic, the lack of leadership may account for the vagueness of role delegations. Troubled relations between the MAs probably made it difficult to establish a clear leader and hierarchy in the clinic. There were also problems with relations between different cadres of staff, leading to poor communication and morale, with workers left feeling underappreciated. This has been evident in this, and other similar studies. [15, 30–32]. The lack of appreciation lead to staff losing motivation and enthusiasm for the job. This is evidenced in the statements from SNs claiming that staff spent clinic time attending to personal business. The combined lack of communication and clear role delegations may have lead to things being overlooked because nobody had taken responsibility for the task. The MAs in the clinic do not seem to demonstrate the use of this colour coding system and therefore showed the least use of the ETAT system, although they did show an understanding of it when interviewed.

Once patients have been directed to the correct person the condition should be classified as described in the IMCI process. However, there was no evidence of this being carried out in the intended manner. Due to the lack of diversity of those who had been given training in this area, the only people capable of carrying this out would be the MAs. Training was a problem under the subtheme of organization. Even though they had undergone the required training there were no resources available to remind the MAs of the classification system which was a problem of tools and technology. This meant they were working from memory which is an inefficient system and resulted in the classifications being ignored. The standard duration of training in the IMCI process is 11 days, with follow up training afterwards. Other studies have shown that this follow up training does not always happen. In a study based in Uganda follow up happened for only 42% of those trained and 69% of staff felt that their training was not adequate [33]. The reasons for the lack of follow up training were largely based around funding problems, for the training and travel to receive it [34]. Even some of those who had undergone the relevant training seemed unclear on the integrated role of IMCI and ETAT, believing the latter to be replacing the former. This lack of clarity meant that even if they had possessed the ability they may not have used it. This demonstrates a lack of clear communication between the clinic and the government (an external environmental factor) to establish the nature of the schemes.

Once disease has been classified the appropriate treatment should be administered. The preferred treatment for each disease classification is part of the IMCI process, however, due to the classification system not being properly implemented here it is unclear whether the treatments provided adhere strictly to those suggested by IMCI or ETAT. As mentioned before treatment is not administered by HSAs and not commonly by SNs working on their own. Therefore, the burden of this role is largely on the MAs. IMCI suggests that once classified the patient should again be prioritized using a colour coded system. This time to determine the level of treatment required. Red suggests referral to a nearby hospital and any required pre-referral treatment. Yellow suggests treating the patient in clinic and giving them specific medical advice. Green indicates the patient can be treated at home, after advice being given to the parent or guardian on treatment and future prevention methods. Although the classification and colour coding have not been adhered to perfectly, the possible treatment outcomes used in the clinic still loosely fit these categories. One of the barriers to proper implementation of the IMCI treatment guidelines is the lack of equipment and medication to carry them out. This was seen in particular in malaria management. Facilities such as microscopy, rapid testing kits and medications were not available to provide the proper diagnosis and treatment. This was also a concern in another study of the IMCI process in Tanzania, where they suggested new guidelines were required in this area as treatment is currently not supposed to be given until a rapid diagnostic test has given a positive result [33]. Lack of equipment is also seen in the referral system. Other studies have reported that lack of access to an ambulance impeded referrals [35, 36], which was also the case here. However, in this particular clinic referrals were also used commonly in an attempt to compensate for the lack of equipment available at the clinic. Patients were then often required to make their own way to the local hospital. Patients found it frustrating when they were not able to receive the care they needed, or had to pay for it themselves. They became disillusioned with the health service which affected their future receptiveness to health education or advice.

Patient advice and education is a tool that was strongly emphasized in the IMCI guidelines. It appeared to be used readily by HSAs and SNs, both within the community and during patient consultations at the clinic. The community aspect of this was not examined in this study but it should be examined more closely in the future. A similar study based in Uganda found that a lack of understanding of the purposes of IMCI among parents and guardians of paediatric patients was associated with malnutrition in the children [37]. Patient education was not displayed by the MAs during their patient interactions. The interactions they had were very brief because of the sheer volume of patients waiting to be seen. This didn’t leave much time for advice. A study looking into IMCI adherence in Tanzania showed how effective time management impacts directly on the workload subtheme [30]. They identified that measures were not taken to control the overcrowding; instead the intense workload was regarded as the norm, severely limiting the time spent on patient care, even during quieter periods. This idea can also apply in this study’s context where a rushed assessment has become routine.

The follow up process suggested in IMCI was variable between members of staff. If follow up appointments were required, the patient would have to present to the clinic as usual and follow the same triaging system when greeted by HSAs. This was an example of where the ET process was focused on more than the directions from IMCI. Due to the loose structure of the referrals it is the parent or guardian’s responsibility to ensure this is carried through. Problems here can be with the attitudes of the guardians. Often they had different beliefs or priorities that may have influenced whether they return. The health education provided by staff at the clinic should have helped to reinforce the importance of following advice or instructions given by staff. There are limits, such as those imposed by the space available in the facility, that hinder the ability to give these health education sessions to all those who may be interested or find it beneficial. These classes were a useful tool but the topic was selected somewhat at random, rather than tailored to the needs of the people there.

Overall neither of the schemes in place at the clinic seem to be fully rolled out and embraced by the members of staff. Elements of each have been exhibited but there seems to be differing levels of dedication to each between the different tiers of staff. Where ETAT has been embraced by the HSAs in particular, it is actually only the triaging portion of this, very little attention was paid to the treatment section at any level. This may be due to the HSAs not being able to administer complex treatments and therefore focus their attention on triaging the patients. Aspects of IMCI, such as the education and immunization process have been embraced by HSAs and SNs but there is no recognition of the classification, treatment or follow up sections. Follow up may be particularly difficult due to the use of Health Passports. Although these do allow patients to feel more involved in the process it also relies on them to keep track of their own health. Particularly in the cases of patients who do not understand the importance or want to have this control it may be very difficult to keep track of who needs a follow up appointment. The MAs have not been seen to particularly present aspects of either of the systems, although they do claim understanding of the ETAT system in particular. This difference of opinion between staff cadres could be due to their different training levels. It has been noted that HSAs in particular don’t benefit from much training and there is no real clarity about what their role within the clinic is. The student nurses are not tied to the clinic so perhaps they are less knowledgeable of the follow up section in particular as they may not be assigned to each clinic long enough to have a long term role in patient care.

The clinic appears to have adopted parts of each of the two guidelines to different degrees, forming their own version of the guidelines. They have selected parts of each guideline that works best for them based on the resources available to them. The SEIPS model here identified barriers to implementing all aspects of the two guidelines, such as the lack of space leading to difficulty running patient education sessions. Identifying these features provides areas to work on to improve integration of these guidelines. Although the information in this study is specific to this clinic it would be interesting to see if the problems faced here are similar to those in other, similar locations.

Undertaking a research study in a primary health care centre posed many difficulties. Recruitment of participants was problematic due to the requirements for English speaking levels. The workload of staff also made it difficult to find time to conduct interviews without impeding day to day running of the clinic. As mentioned in the methods section, the participants were selected by the contact at the clinic prior to the arrival of the researcher. This may have introduced a bias as it is possible that these candidates were selected in order to present a perspective deemed favourable by this contact. However, this selection prerequisite was unavoidable due to the interviews and analysis being carried out in English by English speakers. A diverse cadre of staff were interviewed to provide different perspectives on the clinic and to provide a larger sample size. In this case all participants were interviewed in the same way, perhaps in future research it would be more beneficial to explore each role more thoroughly, in particular the community work carried out by the HSAs. The observations were also carried out within the healthcare facility by the researcher. These were very informal but were intended to see in person the implementation of the guidelines. It is possible that the staff were acting differently to their usual procedure due to the presence of the researcher as described by the Hawthorne Effect [38]. It was hoped to avoid altering the results significantly by making the interviews the main focus of the researcher’s presence in the facility. The researcher was there as a medical student, there for their own learning and not in a position over staff members. They were also present in the facility for long enough that the staff would be used to their presence and hopefully would therefore be acting as they usually would. Completing similar studies on other facilities in Malawi would give a more general picture of the integration of the IMCI and ETAT programmes as the data collected here is very location specific.

## Conclusion

The SEIPS 2.0 framework has brought to light both barriers and facilitators to the integration of the IMCI and ETAT schemes and therefore the efficient running of the clinic. By examining these a clear picture was exposed of how well these have been integrated and areas to focus on to enable further progress. Although there are elements of both sets of guidelines that have been adopted at the clinic there are others that have not. The barriers brought to light here provide an insight into areas to work on to ensure all elements can be used as intended. The framework gave a unique perspective from which to examine the adaptations used to integrate the two systems in place in Malawi and the challenges this collaboration entails.

Understanding the use of a work systems framework will enable future researchers to undertake similar studies in the qualitative field. Exploring the processes used in the primary health care setting provides insight to future progressions to achieving superior management of childhood illness. Based on the results of this study, recommendations have been generated for this particular clinic that could be relevant to other healthcare settings with similar challenges to overcome.

To improve organisational issues specific roles should be allocated to generate accountability amongst staff. Having a clear leader or specific staff members responsible for organizing job aids would improve the availability of resources and dynamics between staff members. Clearer roles in general could help to alleviate some of the workload if all staff member’s skills and experiences are optimised.For example, this would allow more time to ensure the implementation of patient education as advised by IMCI. Regular team meetings could ensure clear communication and cohesiveness surrounding the goals of the clinic. Training should be universal to ensure adherence to guidelines and refresher courses should be available. This would also ensure that all staff members are on the same page about how the guidelines are intended to be used together. The job aids that act as reminders of these guidelines should be made more durable to improve their availability. Referral documentation should be standardized to improve communications between health centres. The availability of resources needs to be addressed to provide more access to medications, diagnostic testing and an ambulance service.

## Abbreviations

ETAT: Emergency Triage, Assessment and Treatment
HSA: Health Surveillance Assistant
IMCI: Integrated Management of Childhood Illness
MA: Medical Assistant
MDG: Millennium Development Goals
MRDT: Malaria Rapid Diagnostic Test
SEIPS 2.0: Systems Engineering Initiative for Patient Safety
SN: Student Nurse
UN: United Nations
UNICEF: United Nations International Children’s Emergency Fund
WHO: World Health Organisation
